# Editorial: Health and nutrition in the first 1000 days of life

**DOI:** 10.3389/fpubh.2025.1761261

**Published:** 2026-01-12

**Authors:** Yaojiang Shi, Qi Zhang, Yiliang Zhu, Jingchun Nie

**Affiliations:** 1 Center for Experimental Economics in Education, Shaanxi Normal University, Xian, China; 2 School of Community and Environmental Health, Old Dominion University, Norfolk, VA, United States; 3 Department of Internal Medicine, University of New Mexico, Albuquerque, NM, United States

**Keywords:** developing country, early life, health and nutrition, human capital, parenting, the first 1,000 days

It is our great pleasure to introduce this Research Topic on *Health and Nutrition in the First 1000 Days of Life*. The first 1,000 days of life, spanning conception to a child's second birthday, is a pivotal window for shaping lifelong health, cognitive development, and human capital. Yet, in developing countries, this critical period is often marred by malnutrition, inadequate caregiving, and structural barriers that perpetuate intergenerational cycles of poor health and poverty. To address these pressing challenges, the Research Topic “*Health and Nutrition in the First 1000 Days of Life*” brings together 13 rigorous studies from diverse developing contexts—including China, Ethiopia, Colombia, Zambia, South Africa, and so on. These studies offer actionable insights into the consequences, determinants, and solutions of early-life nutrition and health challenges, aiming to inform policy, practice, and future research to safeguard this foundational life stage.

## 1 Motivation for the research topic

Malnutrition and health issues remain persistent global crises, with developing regions bearing the brunt of the burden. According to the World Health Organization, nearly half of under-five deaths in low- and middle-income countries are linked to malnutrition, while stunting, wasting, and micronutrient deficiencies impair the development of millions of children annually. The studies in this Research Topic focus on countries facing distinct yet interconnected challenges: Sub-Saharan African nations grapple with high rates of stunting and maternal high-risk fertility behaviors; China confronts rural-urban disparities in dietary diversity and grandparenting practices; Colombia struggles with intersectional inequalities driving infant mortality; and Zambia faces unique nutritional barriers in resource-constrained mining communities, etc. By centering these contexts, the Research Topic fills critical gaps in region-specific evidence, as many global health frameworks often lack granular data on how local cultures, economies, and infrastructures shape early-life nutrition outcomes. The motivation is clear: to consolidate rigorous, context-sensitive research that moves beyond one-size-fits-all solutions and empowers policymakers to design interventions tailored to local realities.

## 2 Contributions of the research topic

The articles in this Research Topic cluster around four interconnected themes, each shedding light on critical dimensions of early-life health and nutrition.

(a) Consequences of Health and Nutrition Problems in the First 1,000 Days

The first group of studies quantifies the far-reaching impacts of suboptimal nutrition and health in the first 1,000 days. Liu et al.'s global analysis reveals that iodine deficiency remains a leading cause of childhood developmental intellectual disability, with low socio-demographic index (SDI) regions like Central Sub-Saharan Africa bearing 368 times the burden of high-SDI countries. Qin et al.'s longitudinal study in rural China demonstrates that dietary diversity directly enhances cognitive development in children aged 6–54 months and non-cognitive skills (e.g., prosocial behavior) in children over 2 years, while reducing illness incidence by 7 days in 2 weeks. Taborda Restrepo's mini-review in Colombia highlights that malnutrition-related infant mortality is deeply intertwined with ethnic marginalization and gender inequalities, with indigenous and Afro-descendant children accounting for 51% of such deaths despite representing only 12% of the population. Together, these studies underscore that early-life nutrition gaps are not merely health issues but drivers of cognitive impairment, developmental delays, and structural inequities.

(b) Barriers and Determinants of Health and Nutrition Problems

The second group identifies the multi-layered barriers to optimal nutrition and health. Qiao et al. find that while China's systematic vitamin D supplementation has reduced deficiency rates to 2.8%, children aged 3–6 years face heightened risk due to reduced sunlight exposure and declining supplementation compliance. Xiong et al.'s predictive model for stunting in Shenzhen, China, pinpoints male gender, mixed/bottle feeding, and limited parent-child reading time as key risk factors, offering a scalable tool for early identification. Seifu et al.'s analysis of 161,179 children across 26 Sub-Saharan African countries shows that maternal high-risk fertility behaviors (early/late childbearing, short birth intervals) increase stunting risk by 2.12% and underweight risk by 2.68%. Li et al. highlight urban-rural disparities in grandparenting in China, with rural grandparents' limited caregiving knowledge linked to higher malnutrition and developmental delay risks. In Zambian mining communities, structural barriers—prioritizing bills over food, limited access to farmers' markets, and overcooking vegetables—drive underweight rates of 20% among under-fives. Wang et al.'s finds that 41.44% of pregnant women in rural China had unintended pregnancies, linked to higher anxiety (OR = 1.96) and stress (OR = 2.15). Unemployed women and those co-residing with mothers-in-law face more risks. These studies collectively show that early-life nutrition challenges stem from a mix of individual behaviors, caregiving practices, and structural inequalities.

(c) Barriers to Parenting Behavior and Public Service Utilization

The third group focuses on factors shaping caregivers' ability to access services and adopt healthy practices. Kasse et al.'s study in Ethiopia finds that only 47.6% of mothers practice key essential nutrition actions (ENA), with secondary education, institutional delivery, and ANC nutritional counseling as critical enablers. Yang et al.'s and Xiang et al.'s studies on public service utilization highlight that peer interaction and health knowledge gaps hinder access to maternal and child health services in rural contexts, while cultural norms and limited awareness prevent caregivers from leveraging available support. These findings emphasize that improving early-life nutrition requires addressing not just what caregivers “know” but also their ability to access services and overcome structural barriers to behavior change.

(d) Economic Evaluation of Interventions

Zhang et al.'s economic evaluation of the NEON program in London's South Asian communities (4–2) provides a crucial evidence base for intervention scalability. The study finds that the participatory learning and action (PLA) intervention—targeting feeding, care, and dental hygiene—costs £439 per mother (face-to-face) and £407 (online), accounting for less than 0.1% of local authorities' child development budgets. This demonstrates that culturally tailored, community-led interventions are financially sustainable, offering a model for developing countries to invest in cost-effective early-life nutrition programs.

## 3 Conclusion

This Research Topic reinforces that investing in the first 1,000 days requires context-sensitive, multi-sectoral action. The studies collectively show that while the consequences of early-life nutrition gaps are severe—from cognitive disability to increased mortality—the barriers are addressable through targeted interventions: scaling micronutrient supplementation, improving dietary diversity, strengthening maternal health services, equipping caregivers with knowledge, and addressing structural inequalities. For policymakers, the evidence underscores the need to prioritize early-life nutrition in national health agendas, integrate nutrition counseling into routine care, and allocate resources to marginalized communities. For researchers, the Research Topic highlights gaps in long-term intervention impacts and the need for more studies on urban-rural and intersectional disparities. Ultimately, the first 1,000 days offer an unparalleled opportunity to break cycles of malnutrition and poverty—one that demands urgent, evidence-driven action to ensure every child has the chance to thrive.

